# Degenerative spine disease: Italian position paper on acquisition, interpretation and reporting of Magnetic Resonance Imaging

**DOI:** 10.1186/s13244-020-00952-w

**Published:** 2021-02-11

**Authors:** Francesca B. Pizzini, Mattia Poletti, Alberto Beltramello, Mario Muto, Alessandra Splendiani, Sara Mehrabi, Giuseppe Costanzo, Vincenzo Vitiello, Antonio Barile, Stefano Colagrande, Giancarlo Mansueto, Stefano Bastianello

**Affiliations:** 1 Department of Diagnostic and Public Health, University of Verona, Piazzale L.A. Scuro, 10, 37100 Verona, Italy; 2 Department of Radiology, IRCCS “Sacro Cuore-Don Calabria”, Negrar, Verona, Italy; 3 Diagnostic and Interventional Neuroradiology, Cardarelli Hospital, Naples, Italy; 4 Department of Biotechnological and Applied Clinical Sciences, University of L’Aquila, L’Aquila, Italy; 5 La Sapienza University of Rome, Rome, Italy; 6 Department of Neurosurgery, San Giovanni Bosco, Naples, Italy; 7 Department of Experimental and Clinical Biomedical Sciences, Radiodiagnostic Unit N. 2, University of Florence-Azienda Ospedaliero-Universitaria Careggi, Florence, Italy; 8 Neuroradiology Department, IRCCS Mondino Foundation, Pavia, Italy; 9 Department of Brain and Behavioral Sciences, University of Pavia, Pavia, Italy

**Keywords:** Degenerative spine, Intervertebral disc herniation, Consensus, Magnetic Resonance

## Abstract

**Objective:**

To promote a better radiological interpretation of spine degeneration, a consistent standardization of the acquisition, interpretation and description of Magnetic Resonance Imaging (MRI) l findings.

**Materials and methods:**

In order to achieve this objective, a consensus among experts in imaging of degenerative spine disease (DSD) from Italian radiological societies (SIRM—Italian Society of Radiology, AINR—Italian Association of Neuroradiology) was achieved. The representatives of the Italian inter-societal working group examined the literature produced by European/American task forces on optimizing the study sequences, classification of degenerative disc changes, spondylo-arthrosis, osteochondrosis, synovial and ligament pathologies of the spinal column, and on canal and foraminal stenosis. The document-resulted from the consensus between experts—was then presented to the scientific societies of Neurosurgery (SINCH) and Orthopedics and Traumatology (SIOT) for their approval.

**Results:**

This position paper presents a proposal for an optimized MRI protocol for studying DSD and provides a glossary of terms related to this pathology and indications on their use. The international terminological recommendations have been translated and adapted to the Italian language and clinical practice and clinical cases have been used to illustrate some of the main classifications.

**Conclusions:**

This revision of international DSD guidelines/recommendations and consensus made it possible to (1) update the nomenclature to international standards and (2) harmonize the MRI protocol and description of radiological findings, adapting both (1, 2) to the Italian context. With this position paper we intend to contribute to an improvement of the communication among doctors and between physicians and their patients as well as the quality of the radiological reports.

Degenerative processes of the spine can cause several health problems [1], including the most common one, low back pain [2].

They derive from the combined action overtime of micro- and macro-mechanical insults, metabolic processes and risk factors (age, sex, work environment, genetics) that affect multiple structures, such as disc-vertebral unit, articular facets, ligaments and spinal muscles.

These osteo-articular and ligamentous elements are part of the Functional Spinal Unit (FSU) [3] which underlies all the degenerative morphological and structural modifications, which progressively involve, first, the disc-vertebral structures of one spinal segment, then the arthro-ligamentous ones at the same level and then, secondarily, involve the adjacent FSU.

Imaging, especially Magnetic Resonance (MR), plays a fundamental role in defining and evaluating Degenerative Spine Disease (DSD), providing the clinician with the necessary support for a correct diagnosis and therapy.

However, in daily clinical practice, the interpretation and description of radiological findings are not harmonized at national level and they are often not updated to international standards [4, 5]. Furthermore, giving the growing use of artificial intelligence software and machine-readable systems and the more and more digitization and sharing of digital data, the implementation of reporting guidelines would facilitate the communication and the sharing of radiological results [6, 7]. So, we proceeded to define a working group (WG) supervised by recognized Italian DSD experts and representatives of the main Italian radiological societies (SIRM—Italian Society of Medical and Interventional Radiology, AINR—Italian Association of Diagnostic and Interventional Neuroradiology).

In the first phase of the work we reviewed the literature produced by European / American task forces [8, 9] which provided indications on how to optimize the study protocol, which nomenclature is the best to use for daily clinical and radiological practice and what are the main updates of the diagnostic criteria of DSD.

In the second phase of the work, a working draft based on these guidelines/recommendations and articles on DSD [3–5, 10–17] was written and shared among the initial panel of experts (AINR, SIRM). The original document was modified through iterative discussion and investigation until consensus was reached on a practical guide for rationalization of.

- (A) MR examination protocol.
- (B) reasoned analytical report.
- (C) study and interpretation of radiological findings.

The last phase of the work involved the submission of the work to the Italian scientific societies of Neurosurgery (SINCH) and Orthopaedics and Traumatology (SIOT) for their approval Therefore, the purpose of this work is to propose a shared and practical guide—based on the review of literature and its translation into Italian scenario—for reaching a reasoned, homogeneous and repeatable reporting that can facilitate the dialogue between clinicians and radiologists and between physicians and patients.

- **(A) MRI technical protocol**

Standard MRI acquisition protocols should be optimized in order to allow the best representation of spinal and paraspinal structures involved in the degenerative pathological processes. Table 1 summarizes the type of sequences useful for each spinal segment, the slice thickness and reference planes and the rationale of their application.

**Table 1:** Summary of the main acquisition parameters (sequences, slice thicknesses and planes) for the study of the spine as a whole and its specific segments

Spinal segment	Sequence/acquisition plan		Slice thickness	Gap	FAT SAT	Notes
CERVICAL	T1/sagittal	TSE	≤ 3 mm	0.5 mm	No	STIR/Dixon reduce metal artefacts T2*/GRE are less sensitive to CSF flow–induced artefacts Oblique acquisition improves the detection and characterization of neural foraminal pathology
	T2 /sagittal	TSE		0.5 mm	No	
	STIR or Dixon/sagittal	TSE		0.5 mm	STIR	
	T2*GRE/axial	TSE		0 mm	No	
	T2/oblique	TSE		0.5 mm	No	
DORSAL/THORACIC	T1/sagittal	TSE	≤ 4 mm	0.5 mm	No	
	T2/sagittal	TSE		0.5 mm	No	
	STIR or Dixon/ sagittal	TSE		0.5 mm	STIR	
	T2*GRE/axial	TSE		1 mm	No	
LUMBAR	T1/sagittal	TSE	≤ 4 mm	0.5 mm	No	T2 axial is preferred to T2*/GRE because there are less CSF flow-induced artefacts at lumbar level T2 coronal provides better evaluation of extraforaminal disc herniation T1 axial is useful for the detection of adipose tissue in the filum terminale
	T2/sagittal	TSE		0.5 mm	No	
	STIR or Dixon/ sagittal	TSE		0.5 mm	STIR	
	T2/axial multistack	TSE		0	No	
	T2/coronal	TSE		0.5 mm	No	
	T1/axial	TSE		0.5 mm	No	
ALL SPINE	T1 Fat Sat/sagittal		2–4 mm			Fat suppression, at least on one plane of acquisition, is required to better evaluate focal contrast enhancement. The same T1-WI with Fat Sat can be acquired pre and post contrast administration to compare CE 2 mm—slice thickness should be considered in the suspect of spinal cord pathology
	T1 Fat Sat/axial					
	T1 Fat Sat/volumetric

- **(B) Guide to the reasoned analytical report of DSD**

Below is reported the suggested format for *drafting the radiological report* of DSD (please see also the sample case and report in “Supplemental Material”). It is divided into 4 points: clinical information, examination techniques, description of findings, their interpretation and conclusions.

- *Relevant clinical indications / information* It is advisable to indicate whether the patient reports (a) only low back pain without radiation or (b) radiated pain (e.g. lumbar sciatica, lumbar cruralgia) and its laterality; (c) sensitivity/motor disorders; (d) the temporal onset of symptoms and their resistance to medical therapy.
- *Examination technique and procedures* The report should include a description of studies and / or procedures performed and any contrast media (CM) used (active substance, quantity), additional medications administered for sedation or for treating any significant adverse reactions or complications associated with drugs or CM.
- *Radiological findings* It is recommended to use the *appropriate terminology* in describing the anatomical and pathological findings and the report of *potential limitations* or limiting factors that may compromise the sensitivity and specificity of the exam. The radiological report should address or answer *any specific clinical questions* or clarify any limiting factors that prevent from answering them. It should also consider previous clinical tests or reports—when relevant and available—for comparison.
- *Impressions/conclusions* they represent a summary of the degenerative processes and of their severity, indicating radiological follow-up or further diagnostic investigation/clinical evaluation, if not yet performed. It should be also considered that clinical-imaging correlation is *fundamental* for deciding the type of treatment—medical, minimally invasive or surgical-.
  Any reactions to a CM administrated should be reported in this final section.

The *radiological report* should be structured considering the spinal functional units as a whole and as singular elements involved in the spinal degeneration process [3]. We therefore propose a reporting scheme (please, see also the scheme in “Supplemental Material”) divided into points, which takes into account the different locations of the degenerative process (1; 2), the secondary radiological findings causing compression (3; 4) of myelo-radicular structures (5), and, finally, the coexistence of paraspinal alterations (6) or incidental findings (7). The description of the findings will then be carried out *according to their clinical relevance and priority*.

1. SPINAL SKELETAL STRUCTURE
  (a) signal or skeletal structural changes.
  (b) curvatures (maintenance, accentuation or reversal of physiological ones).
  (c) vertebral alignment (maintained or not).
2. SPINAL FUNCTIONAL UNITS
  - DISCO-SOMATIC UNITS.
    α) DISC ALTERATIONS.
      (i) pathological changes of signal intensity (SI) and height.
      (ii) morphological changes.
        (x) diffuse displacement—bulging.
        (xx) focal displacement– protrusion or herniation.
        (xxx) coexistence of multiple morphological disc alterations—e.g.
          herniation associated with bulging.
      Description of their location, extent and possible spinal cord or roots compression.
      β) VERTEBRAL BODIES / SUBCHONDRAL BONE MARROW (BM) ALTERATIONS.
  - FACET JOINTS AND LIGAMENTOUS APPARATUS.
3. FORAMINAL STENOSIS
4. SPINAL CANAL STENOSIS
5. CONUS MEDULLARIS AND CAUDA EQUINA Changes of SI and location of conus and cauda (compressive SI changes, clumped and/or abnormal distribution of nerve roots within the dural sac).
6. PARASPINAL SOFT TISSUES AND MUSCLES Abnormalities of soft tissues (e.g. subcutaneous soft tissue edema—lymphedema; adipose infiltration of paravertebral muscles).
7. EXTRASPINAL INCIDENTAL FINDINGS (e.g. aortic aneurysms, liver or kidney lesions, retroperitoneal adenopathy).

- **(C) Clinical-radiological investigation of the most relevant points (1–4), proposed in B.**

1. SPINAL SKELETAL STRUCTURE

It is recommended to describe in the report.

- pathological alterations of the vertebral bone marrow (e.g. primary/secondary tumor or infectious disease), skeletal abnormalities (e.g. height reduction of the vertebral body, asymmetry and/or dysmorphisms of the facet joints).
- accentuation or loss of physiological curvatures.
- metameric misalignment (e.g. spondylolisthesis) on the reference planes (axial and/or sagittal, coronal), also because this may be a direct sign of *radiological instability* or it could be associated to other—indirect-signs of it [3], such as facet fluid collection, synovial cysts, interspinous fluid, facet joint hypertrophy, vacuum degeneration [10]. In case of *spondylolisthesis* [11], it should be specified the severity or-eventually—the degree, according to Meyerding classification, the type (isthmic spondylolysis or degenerative spondylolysis, the latter accompanied by canal reduction) and any worsening of the misalignment during dynamic maneuvers (revealed by flexion and extension MRI).

- SPINAL FUNCTIONAL UNITS
- DISCO-SOMATIC UNITS.
  (α) DISC ALTERATIONS: it is advisable to report any: 
    (i) pathological changes of SI and height of intervertebral disc—indicating T2 signal hypointensity [10], due to dehydration-; any intradiscal gas accumulation (so called vacuum phenomenon, resulting in fluid or T2 hyperintense signal within the disk), or/and annulus fibrous fissures.
    (ii) morphological changes i.e. the displacement of disc material beyond the space of intervertebral disc-delimited cranially and caudally by vertebral bodies and at periphery by apophysis—is defined as:
    (x) *diffuse displacement-bulging* (Fig. 1a), when the disc material extends beyond confines of vertebral endplates for more than 25% or more than 90° of the circumference on the axial plane (> 25%/90°). It may have a symmetrical or asymmetrical extension.

**Fig. 1:**
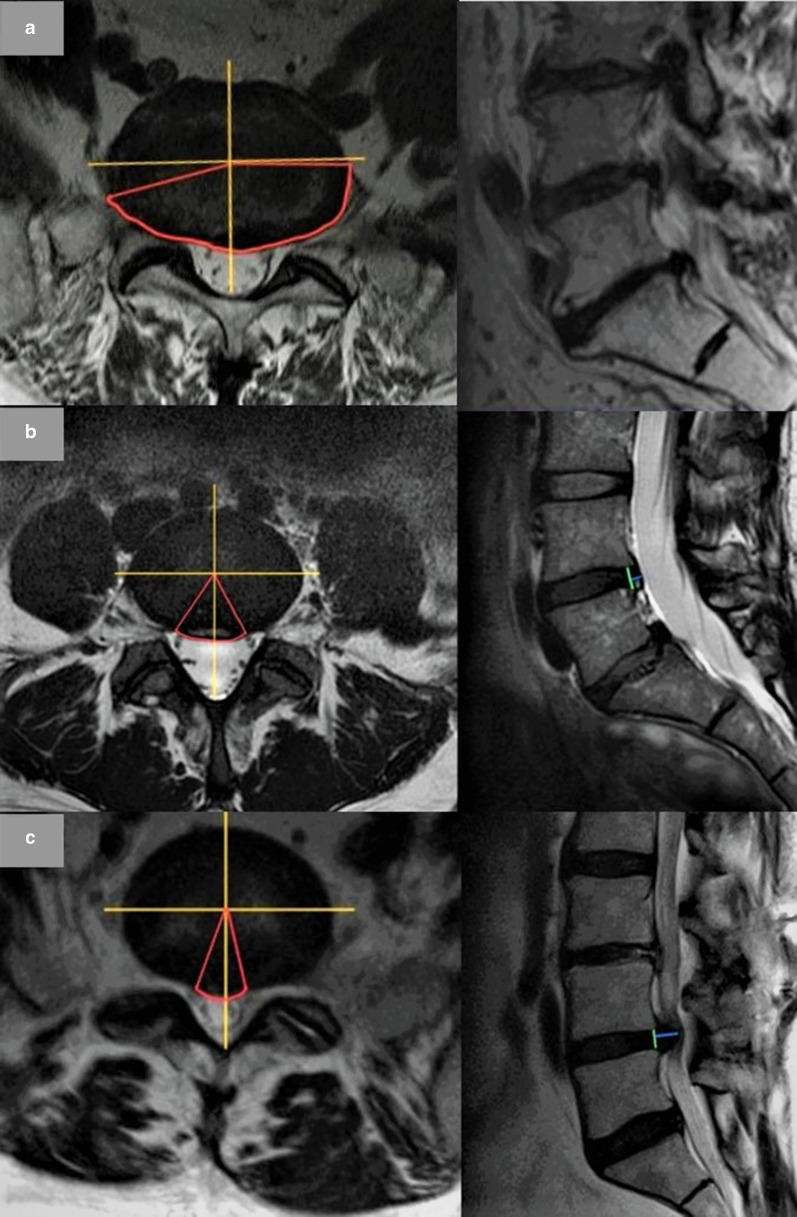
**a** Bulging disc. Wide bulging disc with foraminal extension, more evident on the right. A bulging disc is considered when the extension is more than 25% (> 90°) of the whole disc circumference. Yellow lines show the division in quarters of the disc circumference, red ones the contour of the displaced disc and the angle. **b** Example of disc protrusion. A disc protrusion (in red, the contour of the displaced disc and the angle) is considered when the displacement is less than 25% (< 90°) of the whole disc circumference (in yellow lines the subdivision in quarters of the disc) and the distance between the borders of the displacement (blue line) is less than the distance between the edges of the base of the displacement at the disc space of origin (green line). **c** Example of disc herniation (Extrusion). A herniated disc is considered when the displacement (in red, the contour and the angle) is less than 25% (< 90°) of the whole disc circumference (in yellow lines the subdivision in quarters of the disc) and the distance between the borders of the displacement (blue line) is greater than the distance between the edges of the base of the displacement at the disc space of origin (green line)
    (xx) *focal displacement*, when it is localized for less than 25% or less than of 90° of the circumference on the axial plane (< 25%/90°). Based on their morphological appearance, it can be divided into these subtypes: disc protrusion and disc herniation.
      **Disc protrusion** (Fig. 1b): when the distance between the margins of the disc material dislocated outside the original discal space is *less or equal* than the distance between the edges of the base of the displaced disc material *in all the planes* of acquisition (where the base is measured at the disc space origin).
      **Disc herniation** (disc extrusion) (Fig. 1c): when, *in at least one* plane, the distance between the margins of the disc material dislocated outside the original discal space is *greater than* the distance between the edges of the base of the displaced disc material at the disc space origin.

The report also should include a description of any cranial or caudal migration of disc material (Fig. 2a) and of any loss of continuity with the disc of origin (disc fragment or sequestration, Fig. 2b): in the latter case, it is recommended to specify the disc material location with respect to the Posterior Longitudinal Ligament—PLL (subligamentous, if PLL is intact or extra- or transligamentous, if the PLL is disrupted).

**Fig. 2:**
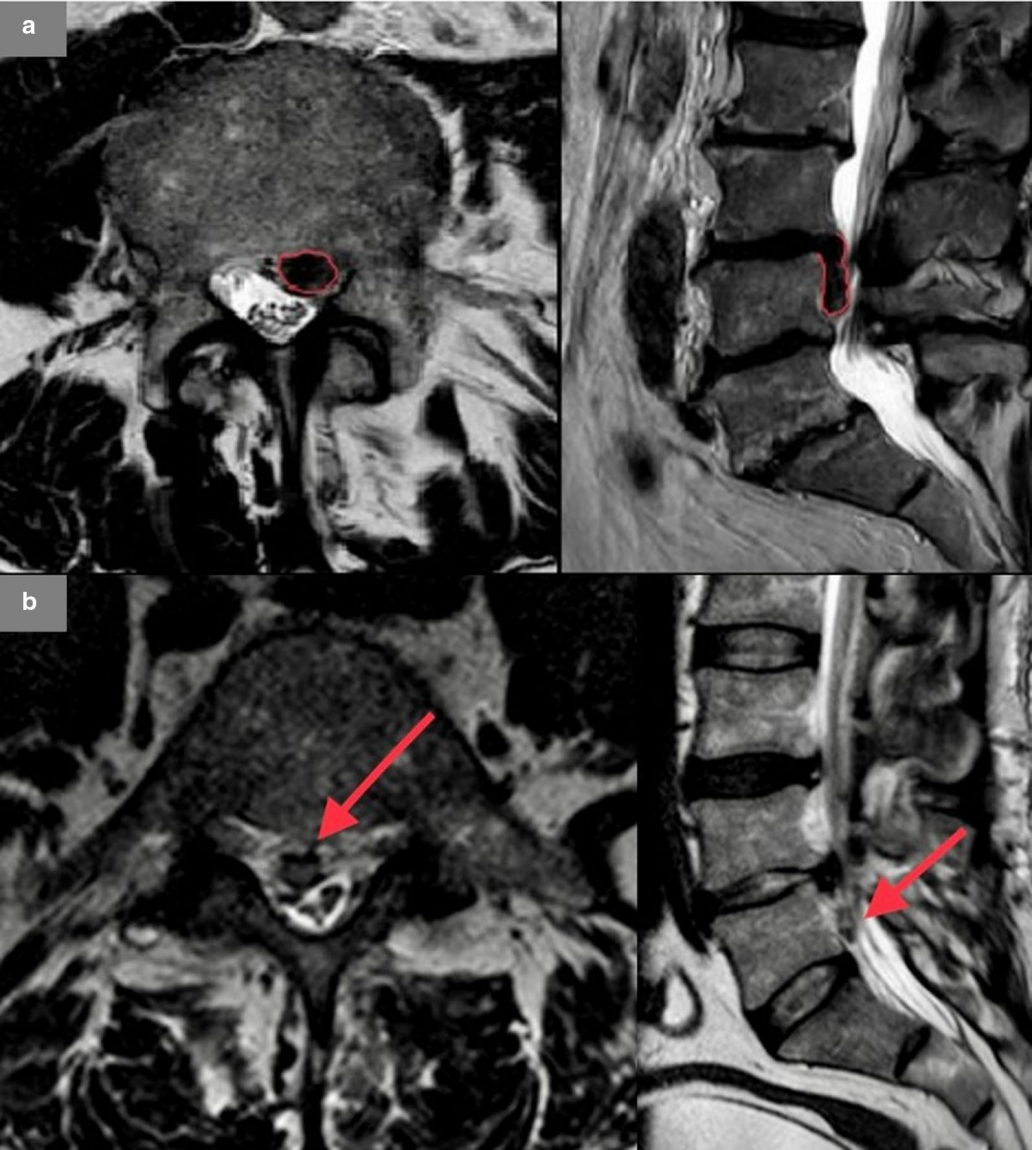
**a** Migration of herniated disc (extrusion with inferior migration of the fragment). A migrated disc is described when extruded material has continuity with the disc of origin. **b** Herniation with sequestration. At L4–L5 large sequestered herniation with right-side caudal migration of the fragment which has no continuity with the disc of origin

In order to describe the location of focal disc displacement, an international anatomical classification has been introduced [3, 8] that takes as reference points some structures of the vertebral body. It provides the recognition of 4 zones (shown on axial plane, in Fig. 3): the central zone, the lateral recess, the foraminal zone and the extraforaminal zone.

**Fig. 3:**
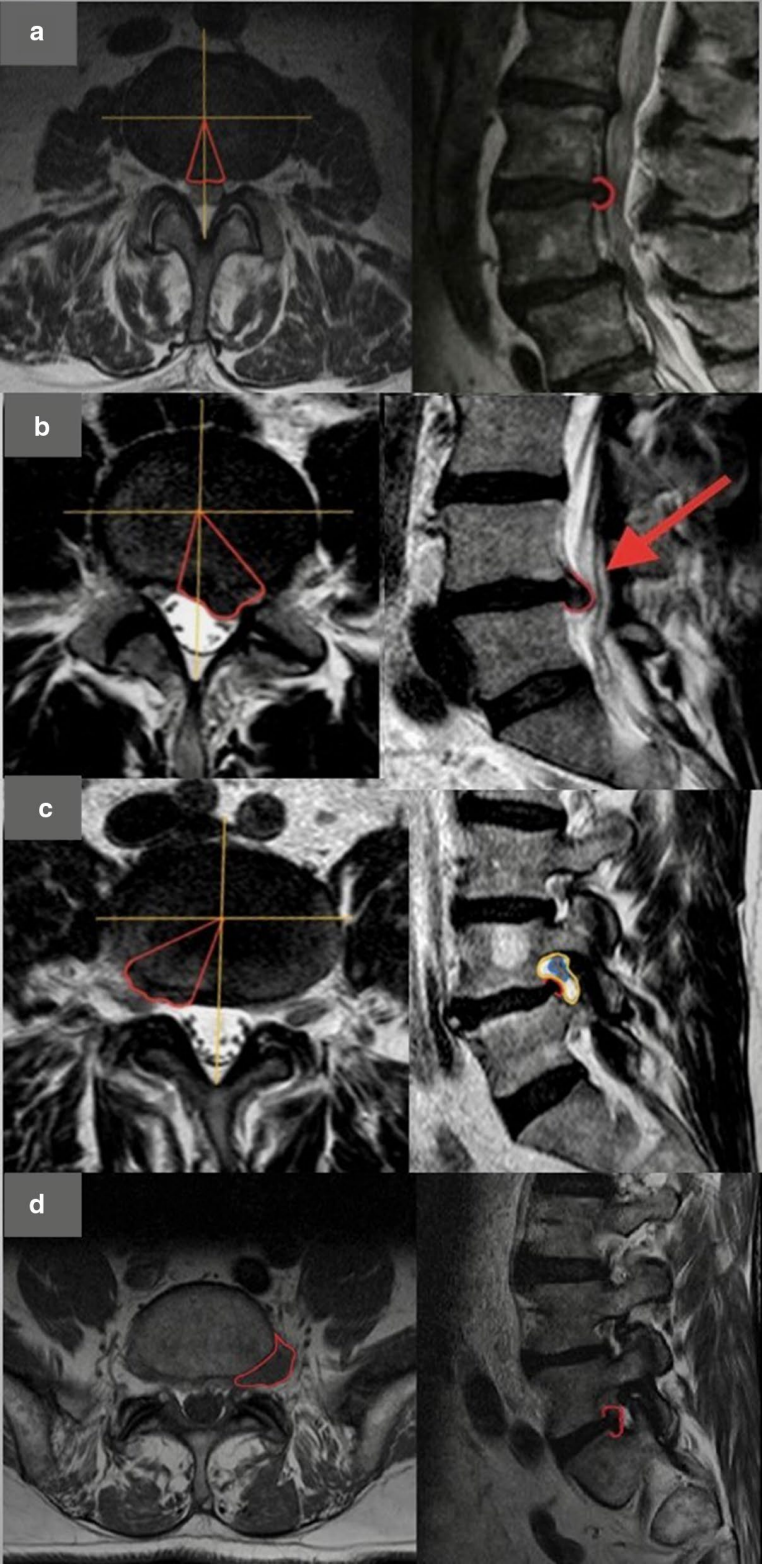
**a** Example of central localization of herniated disc (bordered in red). **b** Example of lateral localization of herniated disc (bordered in red). **c** Example of foraminal localization of the discal protrusion (bordered in red). In sagittal plane, the foraminal space is bordered in yellow and the corresponding nerve root in blue. **d** Example of extraforaminal localization of the herniated disc (bordered in red)

Depending on the zone—or zones—in which the displacement is most localized, we distinguish, respectively, central (Fig. 3a), paramedian (Fig. 3b), foraminal (Fig. 3c) or extraforaminal (Fig. 3d) protrusion/herniations.

It is advisable to specify in the report the (xxx) *coexistence* of herniations/protrusion in one zone with other focal or diffuse disc displacements especially in cases where the latter lead to additional foraminal or spinal canal stenosis.

(β) VERTEBRAL BODIES AND SUBCHONDRAL BM ALTERATIONS

Concerining the degenerative processes of *vertebral bodies*, the report should include a description of vertebral spondylosis (sclerosis and irregularities of vertebral end plates and osteophytosis) and the severity of the findings (minimal, mild, moderate, severe), even without using classifications, such as Kellgren’s one [12].

The description of degenerative findings of *subchondral bone marrow* may be limited to the generic definition of "osteochondrosis"-without specifying Modic’s [13] classification type, if others are the most relevant degenerative pathological findings-.

(b) FACET JOINTS AND LIGAMENTOUS APPARATUS

The report should identify these degenerative findings:

**Arthrosis**—characterized by irregularity, sclerosis and osteophytosis and articular joint space narrowing and synovial thickening-, the presence of synovitis (facet joint fluid collection) and/or synovial cysts (Fig. 4) and the description of the severity of these findings (minimal, mild, moderate, severe).

**Fig. 4:**
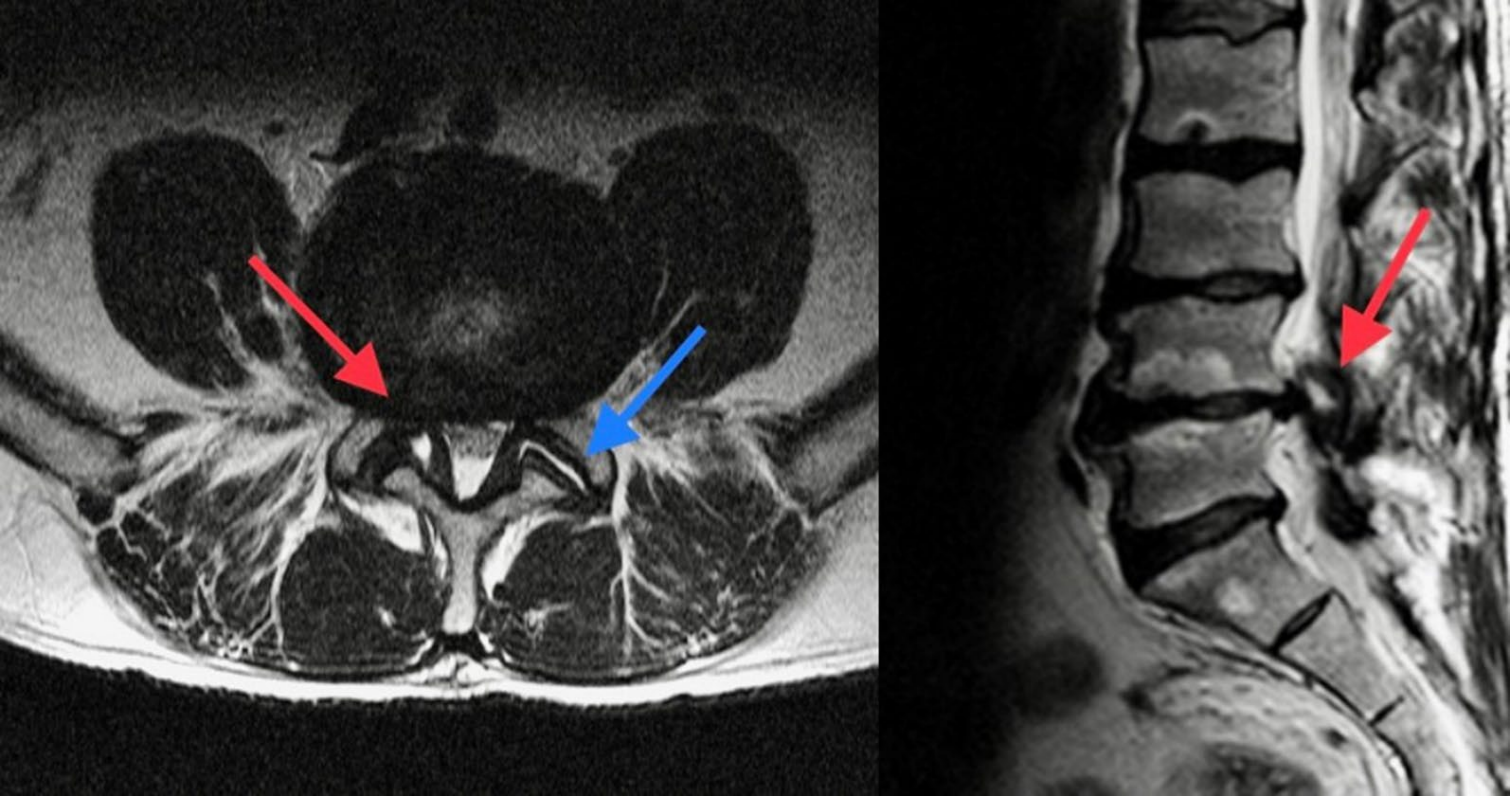
Degenerative changes of the facet joints. Synovial cyst L4–L5 on the right (red arrow on axial and sagittal planes) with spinal canal expansion and lateral recess stenosis. Facet joint fluid collection on the left

**thickening** (or "corrugation") of the **ligamentum flavum** (or yellow ligament). The use of the term "hypertrophy" is strongly discouraged because the degenerative process is not characterized by an enlargement of cellular elements, but by a degeneration of the elastic fibers and an accumulation of collagen due to chronic inflammation; this process determines the corrugation of the ligament and predisposes it to calcification. The thickness of the ligamentum flavum increases with age and varies according to the spinal level. It is advisable to consider as an indicative maximum thickness of more than 4 mm [14] and, regardless this cutoff measure, to report this finding if it is responsible or co-responsible of canal and/or lateral recesses stenosis (point 4 and 5).

3. FORAMINAL STENOSIS

We recommend the following definition of the visual qualitative and quantitative degree of severity of foraminal stenosis [15] (Fig. 5a):

**Fig. 5:**
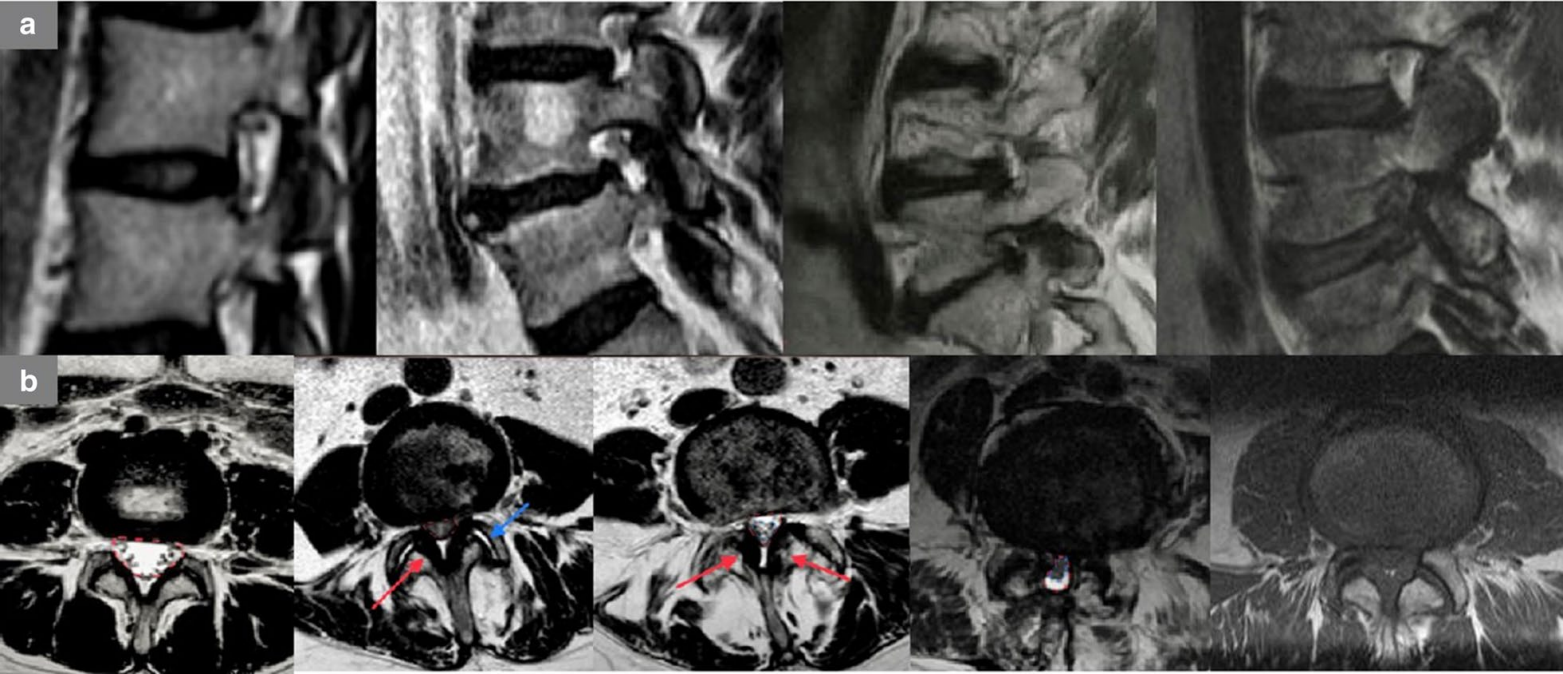
**a** Classification of foraminal stenosis on sagittal T2-WI starting from normal findings (Left) to stenosis (Right): normal width of the foramina; slight stenosis; moderate stenosis; severe stenosis. **b** Classification of spinal canal stenosis on axial  T2-WI starting from normal findings to stenosis. From Left to Right: Grade 0, normal width of the spinal canal, no stenosis; Grade 1, slight stenosis, without significant aggregation of the nerve roots (the reduction in size of the canal is due to bilateral hypertrophy of the ligamentum flavum—red arrow; a bilateral facet fluid joint collection is also associated—blue arrow); Grade 2, moderate stenosis (bilateral hypertrophy of the ligamentum flavum—red arrow. Nerves of cauda equina are aggregated, but cerebrospinal fluid—CSF—is still evident); Grade 3, severe stenosis (entire cauda equina appears as a bundle due to all degenerative processes’ compression, but posterior epidural fat is still visible); Grade 4, extreme severe stenosis (no rootlets and posterior epidural fat are visible)

- Grade 1 (mild stenosis): stenosis < 50%; perineural fat tissue is reduced, but it still completely surrounds the root;
- Grade 2 (moderate stenosis): stenosis > 50%; perineural fat tissue only partially surrounds the root;
- Grade 3 (severe stenosis): complete obliteration of foramen; pinched nerve in the foraminal zone due to extrinsic compression.

4. SPINAL CANAL STENOSIS

It is recommended to explain the degenerative cause(s) leading to canal stenosis (disc displacement—herniation/protrusion/bulging-, facet joint hypertrophy, thickening of ligamentum flavum and spondylolisthesis) and to provide a visual qualitative and quantitative assessment of the grade of severity without any measurements, according to the following scale [16] (example at lumbar level, Fig. 5b).

- Grade 1 (mild stenosis): initial compression and reduction of the dural sac area; cauda rootlets are clearly distinguishable; at cervical/dorsal levels, less than of 50% of cerebrospinal fluid (CSF) obliteration with no spinal cord deformity.
- Grade 2 (moderate stenosis): aggregation of the rootlets of the cauda; CSF film is still surrounding them; at cervical/dorsal levels, more than of 50% of obliteration of CSF surrounding the spinal cord with initial deformity of the cord, but without signal change due to compressive myelopathy.
- Grade 3 (severe stenosis): the rootlets of the cauda appear as a bundle, with no CSF signal around them with posterior epidural fat present; at cervical/dorsal levels, more than of 50% of obliteration of the subarachnoid space surrounding the spinal cord with compression and signal change of the cord.
- Grade 4 (extreme stenosis): no rootlets and posterior epidural fat are visible; at cervical/dorsal levels, complete CSF obliteration and spinal cord compression.

This grading system—along with the clinical scenario—is helpful for surgeons to base their decision making, since severe or extreme severe spinal canal stenosis has been reported to represent an important finding in management of patients [17]. It is advisable to observe and specify the presence of isolated *stenosis of the lateral recess*, as it is responsible for the compression of the adjacent root, thus generating radiculopathy at the inferior level.

## Conclusions

This position paper of the main Italian scientific societies of radiology with the consensus of Neurosurgeons and Orthopedics societies is an important step for improving a common approach to DSD at national level. The revision and discussion of the international guidelines/recommendations and the recent literature on DSD, provided new insights into this pathology and allowed a critical and reasoned proposal in Italian of the most appropriate terms and expressions that should be routinely used. This position paper is the first attempt of consensus for a common interpretation and terminology between radiological and non-radiological specialities and it’s in line with the international standards of reference, with the exception of the definition of disc herniation that we consider a subtype of focal displacement, synonymous of “extrusion” and opposite to “protrusion”. The results of this consensus will impact communication not only of the radiological findings between physicians and doctors-patients, but also the quality of the report, disemboguing terms and suggesting a practical and etiopathological approach. Furthermore, a similar revision of the international recommendations and consensus on interpretation and description of DSD findings—especially the translation of the terminology into each specific language and use—could be also applied to other European countries.

## Supplementary Information

**Additional file 1**. Electronic Supplementary Material.
