# Supplementary material for: Degenerative spine disease: Italian position paper on acquisition, interpretation and reporting of Magnetic Resonance Imaging
Source: Insights Imaging. 2021 Feb 11;12:14. doi: 10.1186/s13244-020-00952-w (PMC7878635; doi:10.1186/s13244-020-00952-w)
Supplement: Supplementary file 1 — Additional file 1. Electronic Supplementary Material. [file 13244_2020_952_MOESM1_ESM.docx]

**ELECTRONIC SUPPLEMENTARY MATERIAL**

**Additional illustration of the reporting scheme**


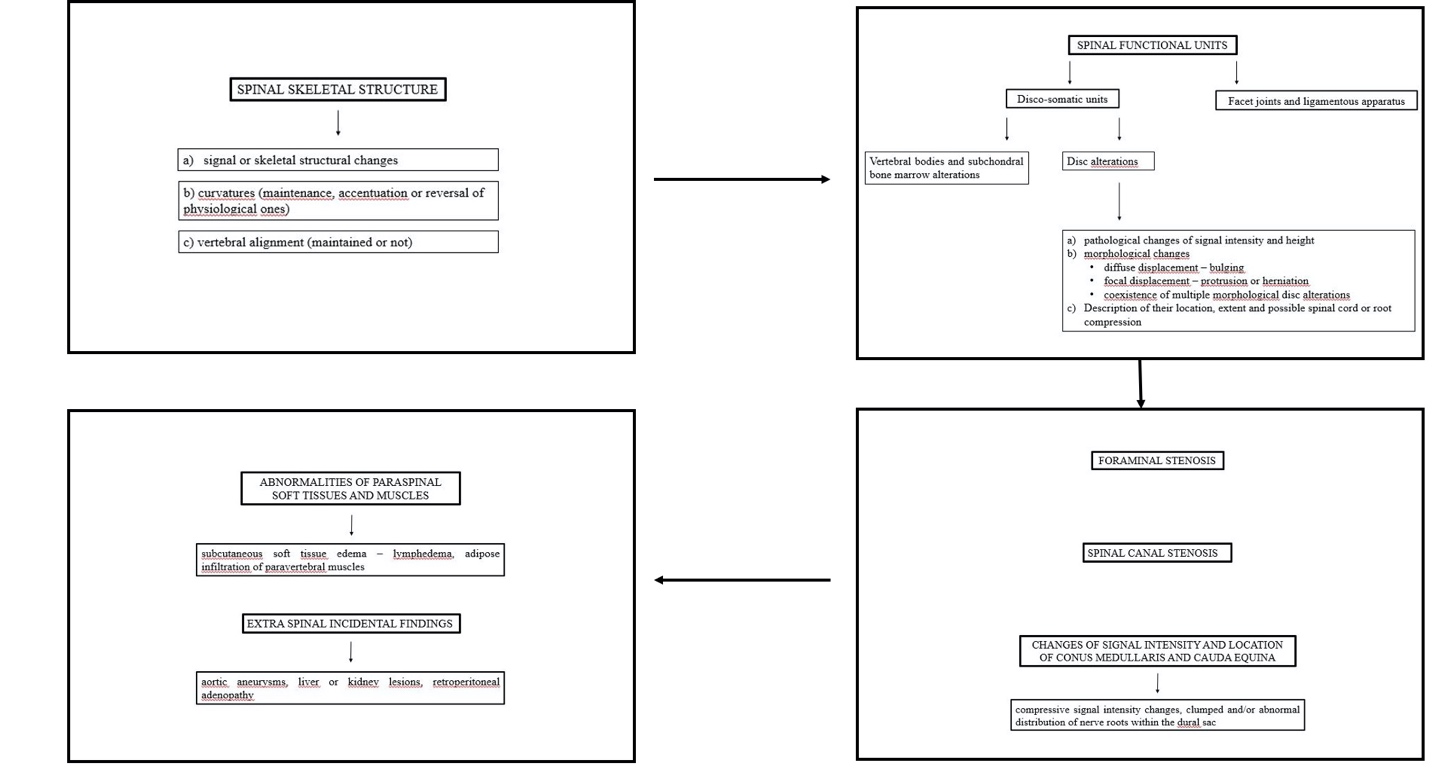


**Sample Case Report**

**
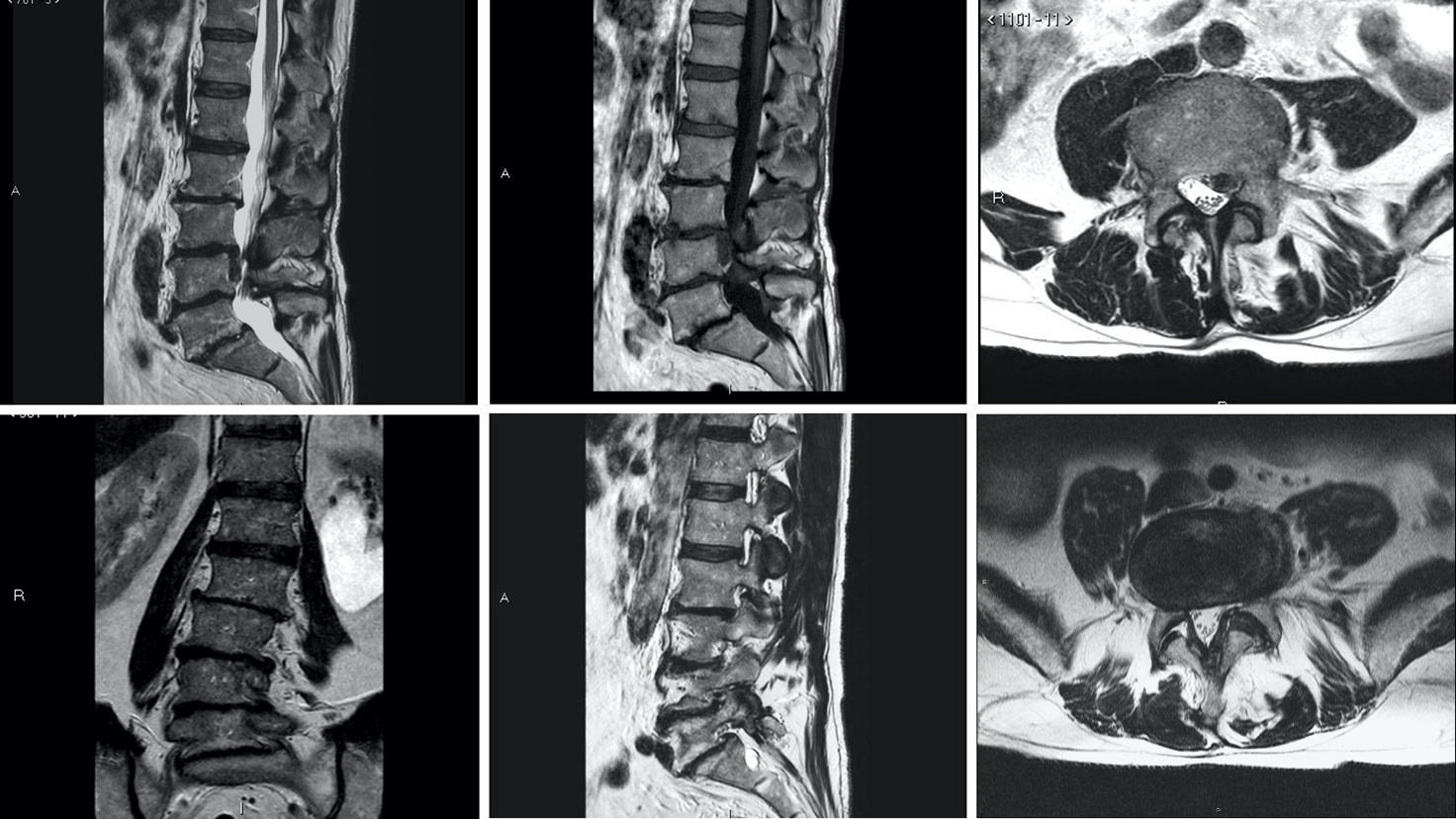
**

***Clinical information*:** 67-years-old male reporting a recent onset -2 weeks- of low back pain with left lumbar cruralgia, in treatment with nonsteroidal anti-inflammatory and skeletal muscle-relaxant drugs - without pain relief after 1 week.

***Technique*:** Sagittal T2, T1, STIR, axial T2 and coronal T2 images.

***Findings*:** lumbar scoliosis with a rightward convexity and decrease of height of the vertebrae and intervertebral discs on the concave side. Mild isthmic spondylolisthesis of L5 on S1 (grade 1 of Meyerding). Subchondral bone marrow changes due to osteochondrosis (Modic 1) at L2/3 and L5/S1.

At L3/4 level, there is a left paracentral inferior migration of herniated disc, compressing the dural sac and the adjacent L4 and L5 roots; there is the coexistence of a disc bulging extending to the vertebral foramina, more prominent on the left.

At the level L1/2, there is a mild disc protrusion, without any foraminal involvement.

At L2/3 and L4/5 there are multiple disc protrusions and osteophytes, compressing the dural sac and slightly narrowing the vertebral foraminal on the left.

At L5/S1 level, there is a posterior disc protrusion, consensual to the spondylolisthesis.

Mild foraminal stenosis is noted on a degenerative basis in the segment between L2 and S1 on the left concave side, which is severe at L4-L5, where it is also seen a facet joint fluid collection on the right.

There is a mild spinal canal stenosis due to multiple factors - spondylosis, discs displacements, arthrosis and thickening of the ligamentum flavum - at all the intervertebral levels between L2 and L5.

Slight adipose infiltration of paravertebral muscles between L4 and S1.

Regular the conus medullaris; mild aggregation of the rootlets of the cauda at the levels of the spinal canal stenosis.

***Impressions / Conclusions:*** left paracentral inferior migration of herniated disc at L3/4, correlated to the clinical onset reported. Degenerative findings at multiple lumbar levels determining mild spinal canal stenosis between L2 and L5 and mild foraminal stenosis (more prominent on the left concave side of the scoliosis L2 and S1 and more severe at L4/5).
